# High Glucose Enhances Cytotoxic T Lymphocyte-Mediated Cytotoxicity

**DOI:** 10.3389/fimmu.2021.689337

**Published:** 2021-06-25

**Authors:** Jie Zhu, Wenjuan Yang, Xiangda Zhou, Dorina Zöphel, Leticia Soriano-Baguet, Denise Dolgener, Christopher Carlein, Chantal Hof, Renping Zhao, Shandong Ye, Eva C. Schwarz, Dirk Brenner, Leticia Prates Roma, Bin Qu

**Affiliations:** ^1^ Department of Endocrinology, The First Affiliated Hospital of USTC, Division of Life Sciences and Medicine, University of Science and Technology of China, Hefei, China; ^2^ Biophysics, Center for Integrative Physiology and Molecular Medicine (CIPMM), School of Medicine, Saarland University, Homburg, Germany; ^3^ Experimental and Molecular Immunology, Department of Infection and Immunity, Luxembourg Institute of Health, Esch-sur-Alzette, Luxembourg; ^4^ Immunology and Genetics, Luxembourg Centre for Systems Biomedicine (LCSB), University of Luxembourg, Belvaux, Luxembourg; ^5^ Faculty of Science, Technology and Medicine, University of Luxembourg, Esch-sur-Alzette, Luxembourg; ^6^ Odense Research Center for Anaphylaxis, Department of Dermatology and Allergy Center, Odense University Hospital University of Southern Denmark, Odense, Denmark; ^7^ INM – Leibniz Institute for New Materials, Saarbrücken, Germany

**Keywords:** high glucose, cytotoxic T lymphocytes, cytotoxicity, glycolysis, glucose uptake, migration, proliferation, Ca^2+^

## Abstract

Cytotoxic T lymphocytes (CTLs) are key players to eliminate tumorigenic or pathogen-infected cells using lytic granules (LG) and Fas ligand (FasL) pathways. Depletion of glucose leads to severely impaired cytotoxic function of CTLs. However, the impact of excessive glucose on CTL functions still remains largely unknown. Here we used primary human CD8^+^ T cells, which were stimulated by CD3/CD28 beads and cultured in medium either containing high glucose (HG, 25 mM) or normal glucose (NG, 5.6 mM). We found that in HG-CTLs, glucose uptake and glycolysis were enhanced, whereas proliferation remained unaltered. Furthermore, CTLs cultured in HG exhibited an enhanced CTL killing efficiency compared to their counterparts in NG. Unexpectedly, expression of cytotoxic proteins (perforin, granzyme A, granzyme B and FasL), LG release, cytokine/cytotoxic protein release and CTL migration remained unchanged in HG-cultured CTLs. Interestingly, additional extracellular Ca^2+^ diminished HG-enhanced CTL killing function. Our findings suggest that in an environment with excessive glucose, CTLs could eliminate target cells more efficiently, at least for a certain period of time, in a Ca^2+^-dependent manner.

## Introduction

Cytotoxic T lymphocytes (CTLs) are the key players to eliminate tumorigenic or pathogen-infected cells (1, 2). CTLs patrol tissues to search for cognate target cells (3–6). Once the targets are identified, killer cells form an intimate contact with the target cells, which is termed immunological synapse (IS) (7–9). To execute their effector functions, CTLs employ mainly two common killing pathways: lytic granules and Fas/Fas ligand (FasL) pathway. Lytic granules contain cytotoxic proteins, such as perforin and granzymes, which can be released at the interface between CTLs and target cells to induce destruction of target cells (10–12). FasL on CTL surface engages with the Fas receptor on target cells to trigger the apoptosis cascades in target cells (13–15).

Glucose metabolism has a central role in regulating effector killing functions of CTLs (16–18). It is known that in absence of glucose, CTLs are not able to exert their effector functions (17). Aerobic glycolysis induced by early T-cell receptor is required for cytokine production in CTLs (19). The availability of glucose modulates IFN-γ production in CTLs (20). Under pathological conditions such as leukemia, glucose metabolism of CD8^+^ T cells is impaired leading to jeopardized cytolytic function of CTLs (21). Notably, enhancement of this impaired glycolysis in CTLs by either checkpoint block or inhibition of autophagy is beneficial for tumor rejection (22, 23). The impact of high glucose on functionality of CTLs remains, however, poorly characterized.

In this work, we investigated the regulation of effector killing function of CTLs by high glucose. We show that stimulating CD8^+^ T cells in high glucose significantly enhances killing efficiency of CTLs without affecting cell proliferation, expression of cytotoxic proteins (perforin, granzyme A, granzyme B and FasL), LG release, or CTL migration. Increase in extracellular Ca^2+^ can diminish this HG-enhanced CTL-mediated cytotoxicity.

## Materials and Methods

### Antibodies and Reagents

The following antibodies or reagents were used: Alexa 488 anti-human CD107a antibody (H4A3, Biolegend), APC-Cy7 anti-human CD3 antibody (HIT3a, Biolegend), BV421 anti-human CD8 antibody (SK1, Biolegend), Alexa 488 anti-human Granzyme A antibody (CB9, Biolegend), Alexa 647 anti-human perforin antibody (dG9, Biolegend), PE anti-human Fas-L antibody (NOK-1, Biolegend), PerCP anti-human CD3 (HIT3a, Biolegend) antibody, Carboxyfluorescein succinimidyl ester (CFSE, Thermo Fisher Scientific), 2-NBDG (ThermoFisher Scientific), Calcein-AM (ThermoFisher Scientific), Protein transport inhibitor (BD), neutralized bovine collagen type I solution (Advanced Biomatrix), Staphylococcal enterotoxin A and B (SEA and SEB, Sigma), CaCl_2_·2H_2_O (Biomol), Paraformaldehyde (PFA, Polysciences), Saponin (Sigma), and streptozotocin (Sigma).

### Cell Culture

Raji cells were cultured in RPMI-1640 medium (ThermoFisher Scientific) containing 10% FCS and 1% Penicillin-Streptomycin. Peripheral blood mononuclear cells (PBMCs) were obtained from healthy donors as described before (24). Primary human CD8^+^ T cells were negatively isolated from PBMCs using Human CD8^+^ T Cell isolation Kits (Miltenyi Biotec) according to the manufacturer’s instruction. Human CD8^+^ T cells were stimulated with CD3/CD28 activator beads (ThermoFisher Scientific) and cultured in DMEM containing normal (5.6 mM, NG) or high glucose (25 mM, HG) (ThermoFisher Scientific) for 3 days if not otherwise mentioned. Since day 2, additional glucose was added into the medium every two days to compensate the consumed glucose along with recombinant human IL-2 (50 ng/ml, ThermoFisher Scientific). All cells were cultured at 37°C with 5% CO_2_. EG7 mouse lymphoma cell line was stably transfected with 4 µg of pCasper-pMax plasmid (25) using SF Cell Line 4D-NucleofectorTM X Kit (Lonza). 48h after transfection, cells were treated with 0.4 mg/ml G418 and 4 µg/ml puromycin in RPMI-1640 (ThermoFisher Scientific) with 10% FCS. Monoclonally expanded EG7-pCasper cells were cultured in selection medium, supplemented with 1% Penicillin/Streptomycin. Flow cytometry analysis revealed 98% of EG7-pCasper cells were fluorescent.

### 2-NBDG Uptake Assay

On day 3 after bead-stimulation, CTLs were loaded with 2-NBDG (120 µM, 5% FCS in PBS) at 37°C for 30 min. Subsequently, loaded CTLs were washed twice in PBS (0.5% BSA) and analyzed immediately by flow cytometry (ex. 465 nm, em. 540 nm).

### Seahorse Assay

Primary human CD8^+^ T cells were stimulated with human CD3/CD28 Activator beads (ThermoFisher Scientific) for 3 days in DMEM containing 5.6 mM or 25 mM glucose (ThermoFisher Scientific). At day 3, CTLs were plated in 96-well XF Cell Culture Microplate in XF Seahorse DMEM medium at a cell density of 3 × 10^5^ cells/well. Following the manufacturer’s instructions (Agilent), the extracellular acidification rate (ECAR) were measured using the XF Glycolytic Stress Test kit.

### Real-Time Killing Assay

The real-time killing assay was carried out as previously described (26). Briefly, Raji cells were pulsed with staphylococcal enterotoxin A (SEA; 1 µg/ml) and SEB (1 µg/ml) at 37°C for 30 min, followed by calcein-AM loading (500 nM, ThermoFisher Scientific) for 15 min at room temperature. Primary CD8^+^ T cells were co-incubated with the target cells with indicated effector to target (E:T) ratios in AIMV medium (glucose 16.09 mM) supplemented with 10 mM HEPES. With addition of CaCl_2_ (1 mM), the respective AIMV medium was pre-equilibrated in the incubator for 24 hours. The fluorescence was measured with a GENiosPro micro-plate reader (TECAN) using bottom reading mode at 37°C every 10 minutes for 4 hours.

### Diabetic CTL Preparation

Male C57BL/6J mice were injected with streptozotocin (STZ) intraperitoneally using a single high dose injection protocol (27). STZ was administered in a concentration of 150 mg/kg body weight diluted in a 0.1 M sodium citrate buffer. Blood samples were taken from the tail vein and glucose levels were tested by standard test strips (Accu-Chek) twice a week. Additionally, overall health status and body weight was monitored and evaluated using a score sheet over the whole observation period. One week after injection, the blood sugar levels were evaluated. Mice with a blood glucose level of more than 280 mg/dL one week after the injection were considered as diabetic and were sacrificed at day 29 after injection. Spleens were removed from mice and kept in sterile PBS on ice until isolation. Splenocytes were isolated with a 40 µm cell strainer (Corning) and erythrocytes were lysed using a hypo-osmolar solution. CD8^+^ T cells were isolated with the Dynabeads™ Untouched™ Mouse CD8 Cells Kit (ThermoFisher Scientific). Purity of isolated cells was evaluated by flow cytometry. Isolated CD8^+^ T cells were stimulated with Dynabeads Mouse T-Activator CD3/CD28 (ThermoFischer Scientific) for 3 days and cultured in AIM V medium (ThermoFisher Scientific) containing 10% FCS, 50 µM ß-Mercaptoethanol and 100 U/ml recombinant human IL-2 (Miltenyi Biotec).

### 3D Killing Assay

For 3D killing assay, the EG7-pCasper cells was used as target cells, which were embedded into collagen (type I bovine, 2 mg/ml) in a 96-well plate. After consolidation of collagen, stimulated mouse CD8^+^ cells were loaded on the top of the collagen as effector cells. The E:T ratio was 10: 1 and all conditions were done in duplicate. The killing was measured by a high-content imaging system (ImageXpress, Molecular Devices) at 37°C with 5% CO_2_ every 10 min for 24 hours. Signal of FRET and GFP channels were analyzed with ImageJ.

### Degranulation Assay

To assess degranulation, SEA/SEB-pulsed Raji cell were settled with CTLs in the presence of Alexa 488 conjugated anti-human CD107a antibody and protein transport inhibitor (GolgiStop, BD). Cells were incubated at 37°C with 5% CO_2_ for 4 hours. Afterwards, the cells were stained with BV421 anti-human CD8 antibody to distinguish target cells from CD8^+^ T cells prior to analysis using flow cytometry.

### Flow Cytometry

To determine proliferation, freshly isolated primary human CD8^+^ T cells were stained with CFSE (1 µM) at room temperature for 10 min prior to CD3/CD28 bead stimulation. To stain perforin and granzyme B, cells were fixed in pre-chilled 4% PFA and permeablized with 0.1% saponin in PBS/0.5% BSA and 5% FCS. Flow cytometry data were acquired using a FACSVerse™ flow cytometer (BD Biosciences) and were analyzed with FlowJo v10 (FLOWJO, LLC).

### RNA Isolation and qRT-PCR

The analysis of mRNA expression was carried out as previously reported (28). Briefly, total RNA was extracted from primary human CD8^+^ T cells after stimulation for 3 days using TRIzol reagent (ThermoFisher Scientific). Subsequently, total RNA was reversely transcribed and the cDNA was used for quantitative Real-time PCR (qRT-PCR). Real-time PCR was performed in a CFX96Real-TimeSystemC1000 Thermal Cycler (Bio-Rad Laboratories) (software: Bio-Rad CFX Manager, Version 3.0). As internal controls, TBP (TATA box-binding protein) and RNA polymerase were used. The mRNA level from the genes of interest is normalized to TBP and RNA polymerase as described elsewhere (29). Primer sequences for the two reference genes *RNAPol* and *TBP* are listed in (29) and for perforin (*PRF1*) in (30). Primer sequences for *GZMA* (NM_006144) are *GZMA_*forw. 5’ TTGGGGCTCACTCAATAACC 3’ and *GZMA*_rev. 5’ TGGTTCCTGGTTTCACATCA 3’, for *GZMB* (NM_00413) *GZMB_*forw. 5’ GAGACGACTTCGTGCTGACA 3’ and *GZMB*_rev. 5’ CTGGGCCTTGTT GCTAGGTA 3’ and for *FASLG* (NM_000639) *FASLG*_forw. 5’ GCACACAGCATCATCT TTGG 3’ and *FASLG*_rev. 5’ CAAGATTGAC CCCGGAAGTA 3’.

### Multiplex Cytokine Assay

Supernatant was harvested at day 3 after bead-activation and kept at -80°C until use. The preset CD8/NK panel (Biolegend) was used. Concentration of each analyte was determined according to manufacturer’s instructions.

### Light-Sheet Microscopy

Visualization of CTL migration using light-sheet microscopy was conducted as previous described (31). Briefly, day 2 after stimulation, the CD3/CD28 beads were removed and the CTLs were loaded with CFSE according to the manufacturer instruction and further cultured in NG- or HG-medium for 24 hours. Then CFSE-loaded CTLs were resuspended in chilled neutralized collagen type I solution (2 mg/ml, Advanced Biomatrix) and polymerized at 37°C with 5% CO_2_ for 40 min followed by 1 hour-recovery in AIMV medium. Migration of CTLs was visualized at 37°C with 5% CO_2_ for 1 hour with an interval of 30 sec using the Z.1 light-sheet microscope (Zeiss). Z-stepsize was 1 μm and 201 slices were obtained for each stack. Using Imaris 8.1.2 (Bitplane), cell trajectories were automatically tracked, the velocity and persistence were analyzed. The CTLs with a track duration less than 10 min were excluded from the quantification.

### Ethical Considerations

Protocols of research carried out for this study with human material (leukocyte reduction system chambers from human blood donors) is authorized by the local ethic committee [declaration from 16.4.2015 (84/15; Prof. Dr. Rettig-Stürmer)]. All individuals fulfilled the donor criteria of the German regulations (32). Informed consent was obtained from all subjects or, if subjects are under 18, from a parent and/or legal guardian. Protocols for the STZ-induced diabetes mouse model for this study is approved by the local regulatory authorities (Animal experiment approval 49/2019, Prof. Leticia Prates Roma). The animal experiments were performed according to local, national, and European Union ethical guidelines.

### Statistical Analysis

GraphPad Prism Software (San Diego, CA, USA) was used for statistical analysis. The differences between two groups were analyzed by paired t-test. For multiple comparisons, two-way ANOVA were performed, followed by Bonferroni test. *P < 0.05; **P < 0.01; ***P < 0.001; ns, not significant. Data are presented as mean or mean ± S.E.M.

## Results

### Glycolysis Is Enhanced in CTLs Cultured in High Glucose

**Figure 1:**
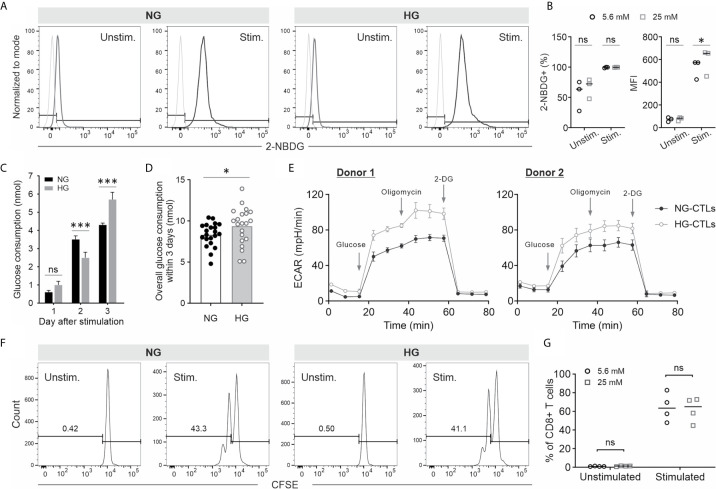
Impact of HG culture on glycolysis and proliferation of CTLs. Primary human CD8+ T cells were stimulated with anti-CD3/CD28-antibody coated beads in medium containing NG (5.6 mM) or HG (25 mM) for 3 days. **(A, B)** Glucose uptake in NG- and HG-cultured CTLs. On day 3, bead-stimulated (stim.) or unstimulated CD8+ T cells (Unstim.) were loaded with 2-NBDG at 37°C for 30 min and analyzed with flow cytometry. FMO for gating is shown in light grey in **(A)**. Fraction of 2-NBDG+ cells and mean fluorescence intensity (MFI) is shown in **(B)**. **(C, D)** Glucose concentration was measured at indicated time points using blood glucose meter (Bayer). Glucose consumption was calculated for 1.5 × 106 CTLs. **(E)** Extracellular acidification rate (ECAR) of CTLs. **(F, G)** Proliferation of CTLs cultured in NG- or HG-medium. Primary human CD8+ cells were stained with CFSE prior to CD3/CD28 bead-activation. The fluorescence was determined with flow cytometry. Results are from three **(A, B)**, twenty-two **(C, D)**, two **(E)** or four donors **(F, G)**. * p < 0.05; *** p < 0.001; ns, not significant.

To analyze whether high glucose affects CTL functions, we used 5.6 mM as normal glucose level (NG) and 25 mM as high glucose (HG), which imitates a severe hyperglycemia condition (33). Primary human CD8^+^ T cells were activated with anti-CD3/CD28 antibody-coated beads in NG- or HG-medium. Given the fact that stimulated CD8^+^ T cells consume significant amount of glucose (34), glucose was added every day to compensate the consumed glucose (**Supplementary Figure 1**). First, we examined the glucose uptake using 2-NBDG, a fluorescent analog of glucose, which cannot be metabolized by glycolytic enzymes. The uptake of 2-NBDG was substantially enhanced in stimulated CD8^+^ T cells compared to their unstimulated counterparts both in NG and HG conditions (**Figures 1A, B**). Although no difference was identified in fraction of 2-NBDG^+^ stimulated CD8^+^ T cells between NG and HG conditions, the mean fluorescence intensity (MFI) of 2-NBDG in HG-CTLs is higher than that in NG-CTLs (**Figures 1A, B**). Since 2-NBDG uptake does not reflect functional change in Glucose transporter 1 (35), we also determined the consumption of glucose on each day after stimulation. We found that although no significant difference was identified for day 1 between HG- and NG-CTLs, for the second day, NG-CTLs consumed more glucose, whereas for the third day HG-CTLs consumed more glucose (**Figure 1C**), which is in good agreement with MFI of 2-NBDG (**Figure 1B**, right panel). Of note, the overall consumption of glucose within 3 days was slightly higher in HG-CTLs than in NG-CTLs (**Figure 1D**).

Next, we examined the impact of HG on glycolysis in CTLs. We used Seahorse Extracellular Flux Analyzer to determine the extracellular acidification rate (ECAR) of NG- and HG-CTLs at day 3 after stimulation. The results show that both glycolysis (between glucose and oligomycin) and glycolytic capacity (between oligomycin and 2-DG) was elevated in HG-CTLs relative to NG-CTLs for both donors examined (**Figure 1E**). This result is in good agreement with our observation that the color of HG medium was more yellowish than NG medium on day 3. Thus, we conclude that glycolysis of fully activated CTLs is enhanced in an environment with excessive glucose.

Compelling evidence shows that deficiency in glycolysis leads to impaired T cell proliferation (36). Here we examined the impact of enhanced glycolysis in CTLs by HG on CTL proliferation. Freshly isolated primary human CD8^+^ T cells were stained with CFSE and then stimulated by anti-CD3/anti-CD28 antibody-coated beads in NG- or HG-medium. The results show that without bead-stimulation, CD8^+^ T cells did not proliferate; with bead-stimulation, proliferation of CD8^+^ T cells cultured in HG stayed in a comparable range as their counterparts in NG (**Figures 1F, G**). This result indicates that HG-enhanced glycolysis in CTLs does not further promote their proliferation.

### CTL-Mediated Cytotoxicity Is Enhanced in an Environment With Excessive Glucose

**Figure 2:**
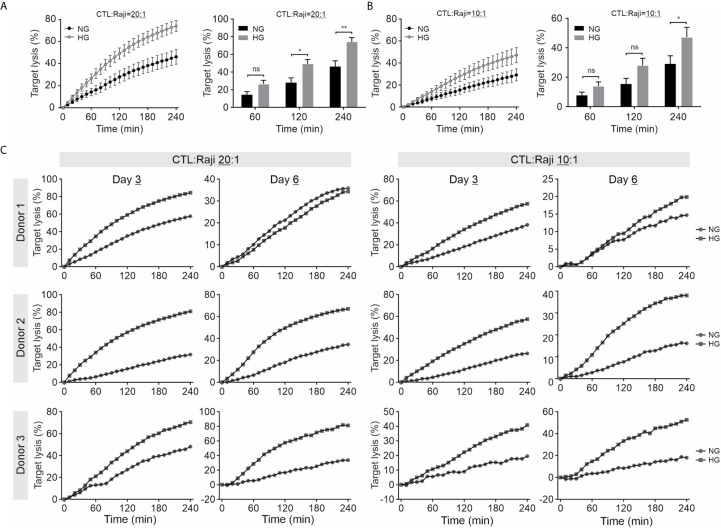
Culture in HG enhances CTL-mediated cytotoxicity. Primary human CD8+ T cells were stimulated with anti-CD3/CD28-antibody coated beads in medium containing NG (5.6 mM) or HG (25 mM) for 3 days **(A-C)** or 6 days **(C)** as indicated. Killing kinetics was determined with the real-time killing assay. SEA/SEA pulsed Raji cells were used as target cells at an effector to target (E:T) ratio of 20:1 or 10:1. Results are from five **(A, B)** or three donors **(C)**. * p < 0.05; ** p < 0.01; ns, not significant.

**Figure 3:**
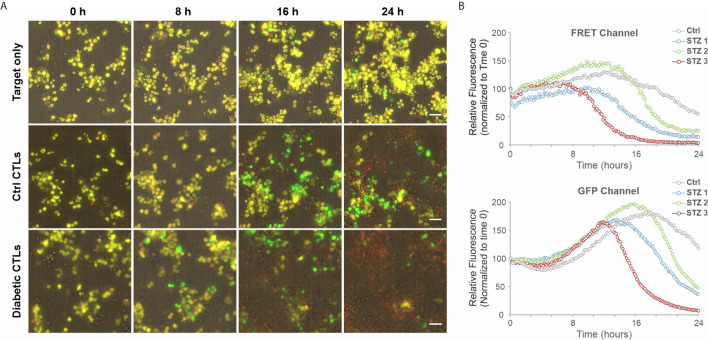
CTLs from diabetic exhibit faster killing kinetics. 3D killing assay was carried out to determine killing kinetics at 37°C with 5% CO_2_ every 10 minutes for 24 hours. EG7-pCasper cells were used as target cells, which were embedded in collagen (2 mg/ml). CTLs were added from top. CTLs from STZ-3 mouse is shown in **(A)**. The quantification of the signal from FRET or GFP channel is shown in **(B)**. Scale bars are 40 μm.

We further investigated the impact of HG on CTL-mediated cytotoxicity. The killing kinetics was determined using the real-time killing assay (26). We observed that cytotoxicity of CTLs is significantly enhanced when cultivated in HG, in comparison with their counterpart in NG on day 3 (**Figures 2A, B**) and day 6 (**Figure 2C**) after CD3/CD28 bead-stimulation. In addition, we also examined cytotoxicity of CTLs from a diabetes mouse model. We visualized CTL-mediated cytotoxicity using a 3D killing assay established in our lab, where the target cells were embedded in 3D collagen matrices and CTLs were added from top. The target cells (EG7-pCasper) stably express an apoptosis reporter pCasper, a GFP-RFP FRET pair, allowing us to distinguish live targets (yellow-colored) from apoptotic (green-colored) or dead (complete loss of fluorescence) targets (**Figure 3A**). In collagen matrices, target cells proliferated during the period of visualization and only a few target cells went apoptotic (**Figure 3A**, Target only, **Movie 1**), indicating that photocytotoxicity of this long-term measurement is negligible. Analyses of conditions with effector cells show that both the control CTLs and the diabetic CTLs induced apoptosis (determined by a drop in FRET signal followed by an increase in GFP signal) and complete destruction of target cells (indicated by an abrupt drop in GFP signal) (**Figures 3A, B**, Movies 2–3*). Remarkably, the CTLs from all three diabetic mice exhibited faster killing kinetics compared to their control counterparts (**Figure 3B**). Together, these results suggest that cytotoxicity of CTLs is enhanced in an environment with excessive glucose.

### HG-Enhanced CTL Is Regulated By Ca2^+^

**Figure 4:**
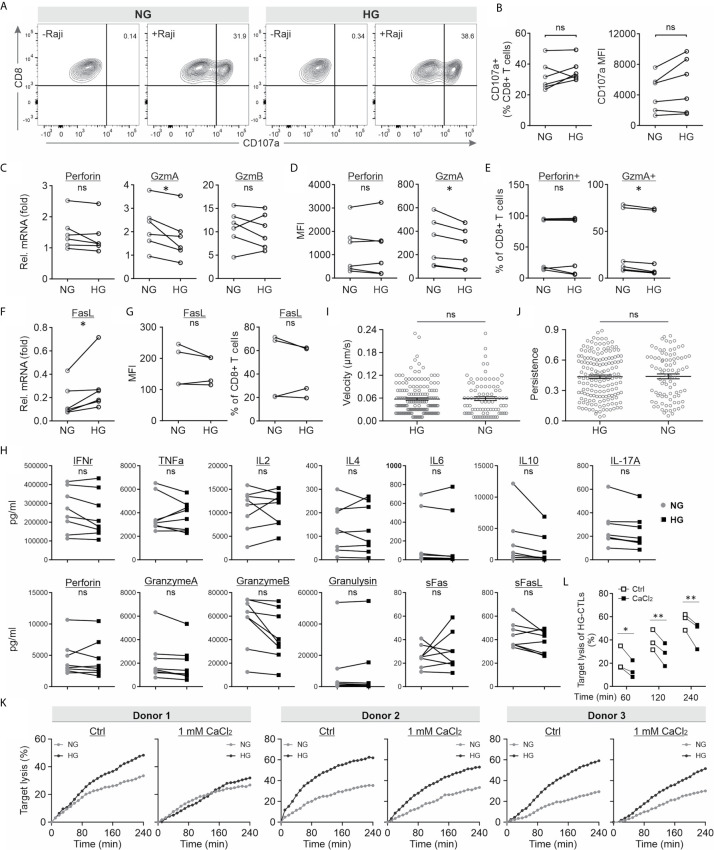
HG-enhanced CTL killing function is regulated by extracellular Ca^2+^. **(A, B)** Degranulation of lytic granules is not influenced by HG. CD 107a degranulation assay was conducted. Raji cells pulsed with SEA/SEB were used as target cells. Results are from six donors. **(C)** The mRNA level was determined by quantitative PCR using TBP and RNA polymerase as internal controls. Results are from six donors. **(D, E)** The protein level was determined using flow cytometry. Mean fluorescence intensity (MFI, **D**) and the positive fraction of CTLs **(E)** are shown. Results are from six donors. **(F, G)** Expression level of FasL at mRNA level **(F)** and at protein level **(G)**. **(H)** Release of cytokines and cytotoxic proteins. Multiplex cytokine assay was performed to determine the concentration of the analytes. Results are from eight donors. **(I, J)** Migration of CTLs is not altered in HG-cultured CTLs. Migration of CTLs was visualized using light-sheet microscopy. Velocity **(I)** and persistence **(J)** were analyzed using Imaris. Results are from two independent experiments and from two donors (88 cells for HG and 162 cells for NG). **(K, L)** Addition of extracellular Ca^2+^ diminishes HG-enhanced CTL killing efficiency. Bead-stimulated primary CD8^+^ T cells and SEA/SEB-pulsed Raji cells were used for the real-time killing assay with addition of CaCl_2_ (1 mM). Target lysis of HG-CTLs at 60, 120 and 240 min were extracted from the kinetics and shown in **(L)**. Results are from two independent experiments and from three donors. * p < 0.05; ** p < 0.01; ns, not significant.

Next, we investigated potential mechanisms how HG could regulate CTL killing efficiency. The lytic granule (LG) pathway is considered as one of the main mechanisms to mediate elimination of target cells by CTLs. Therefore, we investigated the possible impact of HG on release of LG using a CD107a degranulation assay. We found that release of LG upon target-recognition in HG-cultured CTLs is in a comparable range as their counterparts in NG (**Figures 4A, B**). Subsequently, we examined the expression of LG-containing cytotoxic proteins, such as perforin, granzyme A (GzmA) and granzyme B (GzmB) using quantitative PCR and flow cytometry. We found that at mRNA level the expression of perforin and GzmB was not altered, whereas GzmA was even slightly down-regulated by HG (**Figure 4C**). Concomitantly, at protein level, there is no difference identified in perforin but a slight reduction in GzmA in HG-cultured CTLs (**Figures 4D, E**). Since GzmA and GzmB are serine proteases and contribute to target lysis, HG-enhanced CTL killing capacity cannot be attributed to this slight reduction in GzmA expression. The slight GzmA reduction should, if at all, decrease the cytotoxic capacity of CTL but certainly not enhance it. Next, we examined Fas/FasL pathway, another key mechanism employed by CTLs to destroy target cells. Our results show that in HG-cultured CTLs, the expression of FasL was moderately up-regulated at mRNA level (**Figure 4F**), but did not differ at protein level compared to their counterparts in NG (**Figure 4G**). Taken together, our data suggest that both LG machinery and FasL pathway do not contribute to the enhancement of CTL-mediated cytotoxicity by HG.

Although expression of these cytotoxic proteins was not altered by HG, the release could still differ. To examine this hypothesis, we carried out a bead-based multiplex cytokine assay, which enabled us to simultaneously determine up to 13 analytes from same samples. The panel includes six key cytotoxic proteins (perforin, GzmA, GzmB, granulysin, soluble Fas, and soluble FasL) and seven main cytokines produced by CD8/NK cells (IFNγ, TNFα, IL-2, IL-4, IL-6, IL-10 and IL-17A). As shown in **Figure 4H**, no difference was identified in the above-mentioned cytotoxic proteins or cytokines between NG- and HG-CTLs (**Figure 4H**), indicating that the vesicle release machinery in CTLs is not affected by HG. This result suggests that HG-enhanced cytotoxicity of CTLs is not due to change in release of cytokine or cytotoxic proteins.

Migration also plays an essential role in CTL-mediated killing. Generally speaking, higher speed would allow CTLs to find their target cells more quickly, hence increasing the number of killing events in a limited period of time. Therefore, using light-sheet microscopy, we visualized CTL migration in a 3D collagen matrix. The quantification of velocity and persistence shows that there is no significant difference between NG- and HG-treated CTLs (**Figures 4I, J**). These results imply that HG does not alter the motility of CTLs.

Our previous work shows that intracellular calcium in HG-cultured CTLs was decreased upon contact with target cells compared to their counterparts cultured in NG (37). We thus postulated that the enhanced CTL cytotoxicity by HG-culture could be linked to decreased Ca^2+^ influx. To test this postulation, we added extra Ca^2+^ in the medium during the killing assay to elevate the decreased Ca^2+^ influx in HG-cultured CTLs upon target recognition. The real-time killing results show that addition of Ca^2+^ could indeed abolish (Donor1) or diminish (Donor2&3) the difference in killing efficiency between NG- and HG-cultured CTLs (**Figure 4K**). Moreover, we compared the target lysis of HG-CTLs between Ctrl and addition of Ca^2+^ at three time points (60, 120, and 240 min). The statistics show that target lysis of HG-CTLs was significantly diminished with addition of Ca^2+^ (**Figure 4L**). These data suggest intracellular Ca^2+^ as a potential target manipulated by HG to modulate TCR-dependent CTL killing functions.

## Discussion

An increasing number of studies have demonstrated that glycolysis is indispensable for proper killing function of CTLs (38, 39). Up-to-date, most studies investigate functional changes in CTLs in a scenario of abrogation of glycolysis, for example by using the glucose analog 2-Deoxy-D-glucose (2DG) or by excluding glucose in the medium. A recent study also shows that functionally-impaired T cell senescence is correlated with prediabetes and transfer of senescent T cells leads to a deterioration of glucose homeostasis in human and mice (40). Targeting T cell metabolism has been suggested recently as a promising strategy to enhance their anti-tumor function (41, 42). In this work, we investigated the impact of excessive glucose on killing efficiency of CTLs. We found that CTL-mediated killing is enhanced when stimulated and cultured in medium containing HG. Our findings are in good agreement with the previous reports that enhancement of glycolysis in CTLs is beneficial for tumor rejection (22, 23). A recent study has revealed that glucose supplementation substantially improves the survival of virus-infected mice *via* CHOP-mediated tissue tolerance (43). Under this circumstance, enhanced CTL killing capability could also contribute to the ameliorated viral clearance. In addition, we observed that activated CD8^+^ T cells consumed large amount of glucose, supporting the evidence that T cells contribute to glucose homeostasis (44).

High blood glucose level is a typical symptom for diabetes mellitus, which is a major cause of blindness, kidney failure, heart attacks, stroke and lower limb amputation and is the seventh leading cause of death worldwide (45). Type 1 diabetes (T1D) has been identified to be an autoimmune disease for which CTLs play an important role by destroying the insulin-producing pancreatic beta-cells (46). Our findings suggest that high level of blood glucose caused by T1D could potentially further enhance CTL-mediated destruction of pancreatic beta-cells, thus accelerating the progression of the disease. On the other hand, a rich body of literature shows that diabetic patients are more prone to cancer and chronic infections (47–50). In this work, we have examined CTL functions only up to 6 days. Our previous work shows that when co-incubated with the cognate target cells, viability of HG-CTLs did not differ from NG-CTLs at 2 hours, and then fraction of apoptotic CTLs was slightly enhanced in HG condition compared to their NG counterparts (17.8% *vs* 15.1%) (37). It indicates that although cytotoxicity of HG-CTLs is enhanced, but after killing more HG-CTLs undergo apoptosis, which may lead to a decreased number of CTLs in a long run.

In this work, we tried to identify the mechanism upregulating CTL cytotoxicity by HG. We examined CTL proliferation, cytotoxic protein expression, degranulation, migration and cytokine release. None of these factors are altered by HG culture, and they are thus unlikely to be involved in HG-enhanced CTL killing capacity. Viability of HG-CTLs does not explain how cytotoxicity is up-regulated, as our previous work shows that the viability of HG-CTLs did not differ from NG-CTLs at 2 hours after killing, and slightly more HG-CTLs were apoptotic 16 hours after killing compared to their NG counterparts (37). Compelling evidence shows that CTL-mediated cytotoxicity is calcium dependent. More particularly, reduced calcium influx in CTLs upon target recognition by decreasing extracellular Ca^2+^ concentration or down-regulation of Ca^2+^ channel Orai1 leads to an enhancement in CTL-mediated cytotoxicity (28). Hallmarks of IS formation, for example reorientation of microtubule-organizing center, are linked to sustained Ca^2+^ influx (51). In this work, we identified that Ca^2+^ is involved in elevation of CTL killing capacity by HG. Our previous work shows that decreased Ca^2+^ influx upon target recognition was correlated with enhanced CTL-mediated cytotoxicity, and additional extracellular Ca^2+^ led to elevated calcium influx upon target recognition (28). Of note, target recognition-induced calcium influx is decreased in HG-CTLs (37). Concerning the key components of SOCE (store-operated calcium entry) channels (ORAI1, ORAI2, ORAI3, STIM1, and STIM2), in HG-CTLs, ORAI2 and ORAI3 were upregulated, whereas STIM1 and STIM2 were down-regulated at the mRNA level. However, no statistically significant difference of these proteins (ORAI1, STIM1 and STIM2) was identified at the protein level (37). Taken together, the reduced difference in killing between NG- and HG-CTLs by addition of extracellular Ca^2+^ can be explained by the decreased difference in Ca^2+^ influx upon target recognition.

Glycolysis is required for cytotoxic function of CTLs. Inhibition of glycolysis by its inhibitor 2-DG (2-deoxyglucose) reduces cytotoxic capacity of T cells and the expression of key effector molecules such as IFNγ and granzymes (52). It is reported that T cell activation induces rapid aerobic glycolysis *via* PDHK1; and deficiency of PDHK1 leads to decrease in cytokine production but does not affect CD8^+^ T cell-induced target lysis (19). Our results provide the first evidence from the other direction that enhanced glycolysis in HG-CTLs is correlated with increased cytotoxicity.

It is reported previously that cytolytic killing by mouse CTLs is not affected *in vivo* by hyperglycaemia (53). More specifically, the authors used either stimulated OT-I CD8^+^ T cells *in vitro* in medium containing 5.5, 10, or 25 mM glucose or STZ-induced diabetic mouse model (C57BL/6 mice), and the ones with blood glucose > 13.3 mM were considered diabetic. The authors found that glucose levels did not affect glycolysis, proliferation or cytokine release in stimulated OT-I CD8^+^ T cells. Similarly, proliferation, cytokine production, and cytotoxicity of CD8^+^ T cells did not differ between control and diabetic mice. Our work has the same conclusion about the impact of high glucose on proliferation and cytokine production in CTLs as reported by Recino et al. (53). However, there is a contradiction in the results about glycolysis and cytotoxicity in these two studies, which can be well explained by the differences in experiment design between these two studies. In our STZ mouse model, the criterion for diabetes is > 280 mg/dL (corresponding to 15.5 mM) and the mice were sacrificed at day 29. In comparison, Recino et al. used > 13.3 mM and likely sacrificed the mice not later than day 14. Considering the basal level of blood glucose in mice is around 10 mM (the blood glucose of the four mice used in our experiments before injection: 168, 180, 157, and 192 mg/dL, mean = 174.3 mg/dL, corresponding to 9.7 mM), the functional changes in diabetic mouse CTLs can likely be enhanced or become more prominent by more elevated glucose level and longer incubation time like in our case. In addition, the difference between human and mouse CTLs in response to high glucose also cannot be excluded. Of note, the C57BL/6N strain carries a mutation in the nicotinamide nucleotide transhydrogenase (Nnt) gene. This gene encodes a mitochondrial protein and is involved in T cell function (54). We used C57BL/6J strain to avoid this problem.

## Data Availability Statement

The raw data supporting the conclusions of this article will be made available by the authors, without undue reservation.

## Author Contributions

JZ initiated the project and performed glucose measurement in **Figure 1** and **Supplementary Figure 1**, real-time killing assay in **Figure 2**. WY performed degranulation assay, flow cytometry analysis, and multiplex cytokine assay in **Figure 4** and did the quantification analyses for all experiments if not otherwise mentioned. XZ carried out migration experiments, real-time killing assay in **Figure 4**, 3D killing assay in **Figure 3**, and the corresponding analysis. DZ established the EG7-pCasper cell line. DZ and CH isolated and stimulated mouse CD8^+^ T cells. LS-B performed the seahorse assay and the analysis. DD performed 2-NBDG and CFSE experiments in **Figure 1**. CC and LP established the STZ diabetic mouse model. RZ helped with degranulation assay. SY provided expertise in diabetes. ES was involved in qRT-PCR. DB provided expertise in glycolysis and experiment design of seahorse assay. BQ generated concepts, designed experiments, and wrote the manuscript. All authors contributed to the article and approved the submitted version.

## Funding

This project was funded by the Deutsche Forschungsgemeinschaft (Sonderforschungsbereich 1027 project A2 to BQ, TRR219 (M04) to LP), INM Fellow, and HOMFOR2019 (to BQ) and Forschungsgroßgeräte (GZ: INST 256/423-1 FUGG and GZ: INST 256/419-1 FUGG) for flow cytometer and light-sheet microscope, respectively. LS-B and DB are funded by the FNR, respectively by the PRIDE (PRIDE/11012546/NEXTIMMUNE) and the ATTRACT program (A14/BM/7632103).

## Conflict of Interest

The authors declare that the research was conducted in the absence of any commercial or financial relationships that could be construed as a potential conflict of interest.
