# Supplementary material for: High Glucose Enhances Cytotoxic T Lymphocyte-Mediated Cytotoxicity
Source: Front Immunol. 2021 Jun 25;12:689337. doi: 10.3389/fimmu.2021.689337 (PMC8267470; doi:10.3389/fimmu.2021.689337)
Supplement: Supplementary file 1 [file DataSheet_1.pdf]

Supplementary Material

Supplementary Figure legend

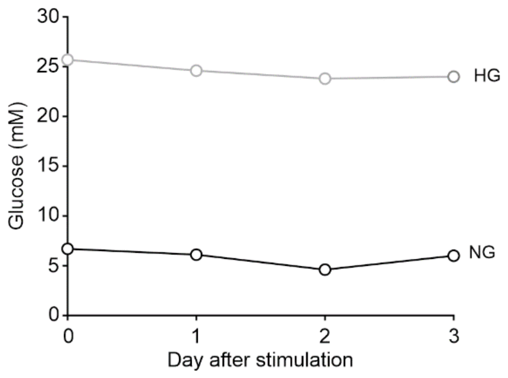

Supplementary Fig. 1 Glucose level after compensation. Primary human CD8$^+$ T cells were stimulated with anti-CD3/CD28-antibody coated beads in medium containing NG (5.6 mM) or HG (25 mM) for 3 days. Consumed glucose was compensated by newly added glucose. Glucose concentration was measured at indicated time points using blood glucose meter (Bayer).

Supplementary Movie legend

Movie 1. Visualzation of EG7-pCasper cells. EG7-pCasper cells were embedded in collagen (2 mg/ml) in a 96-well plate. Signals from FRET channel and GFP channel were acquired with a high-content imaging system (ImageXpress) with an 20× objective at 37ºC with 5% CO_2 every 10 minutes for 24 hours.

Movie 2. Determination of killing kinetics of control mouse CTLs in 3D. EG7-pCasper cells were embedded in collagen (2 mg/ml) in a 96-well plate. Stimulated CTLs (day 3) from the control mouse were added after consolidation of collagen with an E:T ratio of 10:1. Signals from FRET channel and GFP channel were acquired with a high-content imaging system (ImageXpress) with an 20× objective at 37ºC with 5% CO_2 every 10 minutes for 24 hours.

Movie 3. Determination of killing kinetics of diabetic mouse CTLs in 3D. EG7-pCasper cells were embedded in collagen (2 mg/ml) in a 96-well plate. Stimulated CTLs (day 3) from a diabetic mouse (STZ3) were added after consolidation of collagen with an E:T ratio of 10:1. Signals from FRET channel and GFP channel were acquired with a high-content imaging system (ImageXpress) with an 20× objective at 37ºC with 5% CO_2 every 10 minutes for 24 hours.
